# Molecular identification, *in vivo* and *in vitro* activities of *Calvatia gigantea* (macro-fungus) as an antidiabetic agent

**DOI:** 10.1080/21501203.2019.1595204

**Published:** 2019-03-31

**Authors:** Omonike O. Ogbole, Abraham O. Nkumah, Augusta U. Linus, Mofolusho O. Falade

**Affiliations:** a Department of Pharmacognosy, Faculty of Pharmacy, University of Ibadan, Ibadan, Nigeria; b Cellular Parasitology Programme, Cell Biology and Genetics Unit, Department of Zoology, University of Ibadan, Ibadan, Nigeria

**Keywords:** Diabetes mellitus, antidiabetic, alpha amylase, cytoxicity, antioxidant, *Calvatia gigantea*, MTT

## Abstract

Mushrooms are cherished as sources of food, nutrients and medicine. Inadequate data on the identity and medicinal properties of many wild Nigerian mushrooms has limited their utilization. This work was carried out to identify and authenticate a puffball mushroom using molecular tools and investigate its antidiabetic properties. Taxonomic guides were employed in morphological identifying the mushroom as Lycoperdon umbrinum, methanol extract of fruiting bodies was evaluated for antidiabetic activity using in vitro α-amylase assay and in vivo activity in the alloxan-induced diabetic rat model. The macro fungus was identified using Internal Transcribed Spacers (ITS) sequence analysis after which sequences generated were compared using the basic local alignment search tool (BLAST) at NCBI GenBank. In the acute in vivo test, the 400 mg/kg dose showed the best activity with percentage reduction in blood glucose 29.3%, compared with 5 mg/kg glibenclamide at 15%. The in vitro assay established that the extract possessed potent activity with IC50 of 0.46 µg/mL compared to its DCM, butanol fractions and acarbose (IC50 5.3 µg/mL, 5.6 µg/mL, 45 µg/mL) respectively. BLAST analysis revealed the mushroom (accession number, KRO78278.1) to show 98% identity to Calvatia gigantea. The study established the identity of this mushroom and confirmed its antidiabetic activity.

## Introduction

Various studies have shown that some mushrooms are consumed as food and may have potential to lower elevated blood sugar (Valverde et al. 2015; Zhang et al. 2016). Mushrooms have a long history of having been employed by humans as medicines for thousands of years (Ferreira et al. 2010; Valverde et al. 2015; Zhang et al. 2016). Because they have been demonstrated to affect one or more target functions in the body, leading to either an improved state of health and well-being and/or reduction of risk of disease, they are referred to as functional foods (Guillamon et al., 2010, Wang et al. 2017, Vamanu, 2018). Of the 14,000 to 15,000 species of mushrooms in the world around 700 of them have known medicinal properties (Lurie et al. 2009; Li et al. 2018). Edible mushrooms have higher protein contents and minerals and contain less fat but are rich in vitamins B, D, K and sometimes vitamins A and C (Wang, Zhang et al. 2014, Huang, Cai et al. 2016). Several authors have indicated that edible mushrooms are highly nutritional and compared favourably with meat, egg and milk. Furthermore, chemical analysis shows that more than one third of the iron in the mushrooms is in available form and they are increasingly being recognised as one of the important food items for their significant roles in human health, nutrition and diseases (Kumari, Reddy et al. 2011, Zeng, Suwandi et al. 2012, Teke, Kinge et al. 2018)

Many medicinal mushrooms have been found to be suitable for diabetic and heart patients due to low starch and low cholesterol content. Several mushroom species have been reported to be effective for both the control of blood glucose levels and the modification of the course of diabetic complications (Wasser 2011, 2014; D DE et al. 2012; Wu and Xu 2015; Alam et al. 2016; Friedman 2016; Zhang et al. 2016). This is because they are known to contain bioactive components that help with the proper functioning of metabolic organs such as the liver, pancreas and other endocrinal glands, thereby promoting formation of insulin and related hormones which ensure healthy metabolic functioning (Calvo et al. 2016, Amandip Kaur et al. 2015). Mushrooms contain polysaccharides such as beta glucans which can restore the function of pancreatic tissues eventually triggering increased insulin output by β – cells, thus leading to decrease blood glucose levels. Beta glucans have been shown to improve the sensitivity of peripheral tissues to insulin (Rondanelli et al. 2009; Rop et al. 2009; D DE et al. 2012; Chen et al. 2013; Sari et al. 2017)

In Nigeria puffball mushrooms popularly known as ‘Isu or Iso aparo in Yoruba language are part of culinary delicacies with several of the species of puffball mushrooms consumed locally (Oso 1977). Puffball are known especially for their nutritive component and a lot of them have also been reported for biological activities including blood glucose lowering activities (Kivrak et al. 2014; Lee et al. 2017, 2018; Ye et al. 2017). However, accurate identification of these local mushrooms is lacking. The need for unambiguous identification of mushrooms is essential if they are to be maximally utilised as food alternatives and as sources of antidiabetic molecules. Traditional methods of identification of mushrooms based on morphology have been wholly inadequate. Thus, newer DNA sequence methods of identification have offered better discrimination of mushroom species (Jo et al. 2014; Lallawmsanga et al. 2016; Sugawara et al. 2016). Molecular markers, especially DNA markers have been found to be quick and reliable in establishing the identities of mushroom collected from the wild and are helpful in taxonomy (Jo et al. 2014; Khaund and Joshi 2014, 2016; Gawlikowski et al. 2015; Karun and Sridhar 2017). Consequently, this work was carried out to identify and authenticate a puffball mushroom collected in Ibadan, Nigeria, using molecular tools and in addition, to investigating its antidiabetic properties.

## Materials and methods

### Collection of mushroom

The puff ball mushroom was collected from the University of Ibadan botanical garden. The mushroom fruit body was dried and maintained in a desiccator until needed for use. The morphological identification was carried out by mycologist from the Department of Botany and Microbiology, University of Ibadan, where voucher specimens were deposited.

### Extraction of DNA from mushroom sample

Total genomic DNA was extracted from collected mushroom using a plant/fungi DNA isolation kit (NORGEN BIOTEK CORP) according to manufacturer’s instructions. Briefly, the purified genomic DNA from DNA extraction was stored in buffer at – 20°C until required.

### PCR amplification of the ITS region

The entire region of the rDNA of the mushroom sample denoted LUB approximately 670 bp was amplified by PCR using primers ITS 1 (TCCGTAGGTGAACCTGCGG) and ITS 4 (TCCTCCGCTTATTGATATGC). The reaction mix was made up of a total volume of 25 μl, composed of 23 μl of Taq polymerase “Ready to Go” mixture (Pharmacia, Sweden) with 0.2 μl of each primer (100 pM) and 2 μl of DNA template solution. The following thermocycling conditions was used for PCR reaction on a GenAmp PCR System 2400, Perkin-Elmer, USA: 30 cycles of denaturation at 95°C for 30 s; annealing at 50°C for 1 min; and extension at 72°C for 1 min. The amplification products were gel purified and electrophoresed on ethidium-stained agarose gel (0.7%). DNA sequencing was performed using the same primer pair as used previously (ITS 1and ITS 4) in an Applied Biosystem DNA Analyzer (USA).

### Sequence alignment

Alignments were performed using Clustal W (Thompson et al., 1997). The aligned sequences were corrected manually. DNA sequence data were analysed to provide pairwise percentage sequence divergence. The data obtained from the sequence alignment were used to plot a tree diagram using Molecular Evolutionary Genetics Analysis (MEGA 4 Software). The bootstraps varied from 75 to 100. The Neighbor Joining (NJ) method was used (Tamura et al. 2013)

### Preparation of mushroom extract

The powdered mushroom (2.5 kg) was macerated exhaustively in absolute methanol with periodic stirring at room temperature for 72 h. The mixture was filtered using muslin filter attached to a suction pump. The filtrate was concentrated *in vacuo* at 40°C and stored in the refrigerator at 4°C until needed for analysis.

### Cytotoxicity assays

#### Brine shrimp assay

This assay was carried out using method McLaughlin (1998). Eggs of *Artemia salina* (brine shrimp) were obtained from the Department of Pharmacognosy, University of Ibadan. They were hatched in natural sea water obtained from Suntan Beach, Badagry, Lagos, Nigeria and incubated for 48 h in 3.8 g/L sea water. 3 mg of extract was made up to 1 mg/mL in sea water and diluted in ten-fold serial dilutions ending with five concentrations. A suspension of nauplii containing 10–15 shrimp (100 µL) was added to each well, covered and incubated at room temperature (25–29°C) for 24 h. After 24 h, plates were examined under binocular stereomicroscope and the number of dead (non-motile) nauplli in each well were counted. This experiment was done in triplicate. Analysis of data was performed using GraphPad prism to determine the lethal concentration.

#### MTT colorimeter assay

The MTT colorimetric assay was used to evaluate the reduction of viability of cell cultures in the presence and absence of the extracts. The ability of the mushroom extract to be cytotoxic was measured using the tetrazolium dye (MTT), which is metabolized by mitochondrial enzymes of viable (surviving) cells to an insoluble, colored formazan product. The level of metabolism that occurs in the individual well of the 96-well microtitre plate is dependent on the number of healthy viable cells present (Mosmann 1983).

#### In vivo animal study

For animal experiments male Wistar albino rats weighing 150–200 g were obtained commercially and maintained in the animal house at the Physiology Department University of Ibadan. Animals were fed with standard pellet and water given *ad libitum* and acclimatised for three weeks before used for experiments. This study was conducted according to the principles of the Declaration of Helsinki.

Diabetes mellitus was induced intraperitoneally in the experimental rats by the administration of diabetogenic dose of alloxan monohydrate (120 mg/kg body weight). The animals were kept under observation and after 72 h blood glucose was measured by using a One-touch glucometer. The diabetic rats (glucose level > 200 mg/dl) were separated and divided into six different groups of six animals per group.

### Experimental design

Rats were divided into six groups comprising of six animals per group.

Group A: diabetic induced rats treated with 100 mg/kg extract

Group B: diabetic induced rats treated with standard drug glibenclamide

Group C: diabetic induced rats treated with 200 mg/kg of extract

Group D: diabetic induced rats treated with 400 mg/kg of extract

Group E: diabetic induced untreated rats

Group F: normalglyceamic rats administered water and food *ad libitum*)

### Alpha amylase inhibitory assay

The α-amylase inhibitory activities of crude and partitioned fraction were determined based on the modified method of using colorimetric assay (Bernfeld 1955; Aiyer 2005) and acarbose was the reference compound.

### Statistical analysis

The results were expressed as mean ± standard error of the mean (SEM). The statistical significance of the differences was analysed by one-way ANOVA. P < 0.05 was considered significant.

## Results and discussion

**Figure 1.**
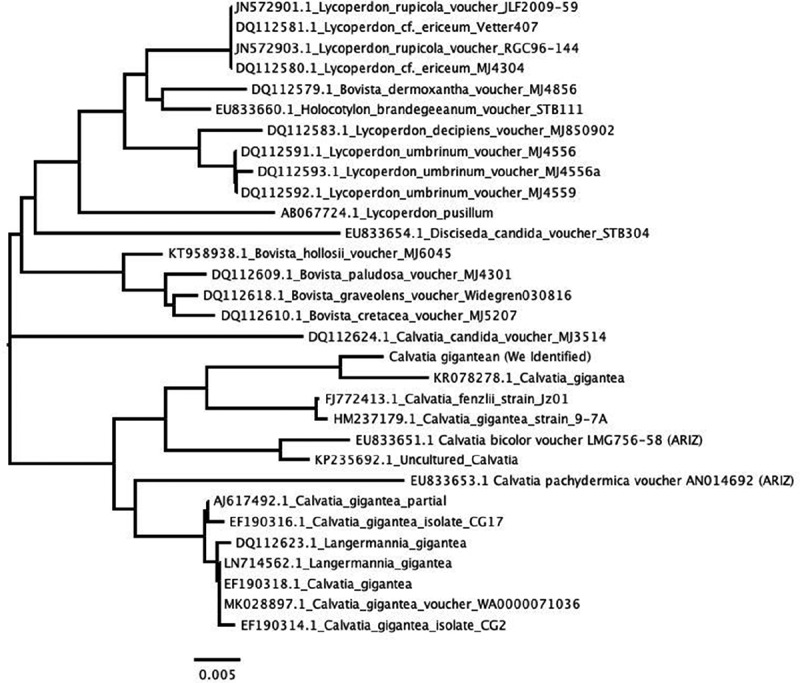
Phylogenetic analysis based on rDNA sequence of *Calvatia gigantea* obtained in this study and reference sequences from GenBank.

The rise in the prevalence of diabetes mellitus and its associated health cost has increased interest in alternative therapies (MEZQUITA RAYA and REYES GARCIA 2014; Coda et al. 2018). Mushrooms have long been associated with humans and provide profound biological and economic benefits. Historical records have provided information on the consumption of wild mushrooms by man for their taste and pleasing flavour (Patel and Goyal 2012; Valverde et al. 2015; Ullah et al. 2017). Morphology and physiological parameter such as appearance, colour, dimension, spores and form of the fungus on media, with other environmental growth preferences is not sufficient for the identification of macrofungi. However, molecular based tools offer better alternatives for accurate identification (Akram et al. 2012; Al-Habib et al. 2014; Wang et al. 2017) of wild medicinal and edible mushrooms, which will help in proper documentation and effective exploration. We have used the Internal transcribed spacer (ITS) sequence for the molecular identification of *Calvatia gigantea*. BLAST analysis result from GenBank showed that the ITS sequence of the mushroom sample (LUB) matched *Calvatia gigantea* with accession number KRO78278.1 (Figure 1). This contradicts the earlier morphological identification as a Lycoperdon species. This observation may be attributable to both Calvatia and Lycoperdon species belonging to the puffball family hence the possibility of substituting one for the another when morphology is sole means of identification.

The Brine shrimp lethality assay is considered as a rapid preliminary assay for screening for the presence of biochemical activity of natural products and was used to determine the toxicity of the crude extract (Meyer et al 1982). This test is based on the lethality of the crude extract against *Artemia salina* nauplli based on the classification of cytoxicity of extract on brine shrimp according to methods established by Meyer and colleagues, *C. gigantia* was considered not cytotoxic having an LC_50_ > 1000µg/mL (Table 1). This was further confirmed by an MTT colorimeter assay against Vero cells that shows *C. gigantea* nontoxic with CC_50_ of 337.4, as compared to cyclophosphamide with CC_50_ of 8.71. Most species of the *Calvatia* genus generally known as giant puffballs are known to be edible, they are used as sources of traditionally nutritional food especially before spore maturation (Eroglu et al. 2016; Lee et al. 2017, 2018), hence their non toxicity in our assays further establish their edibility.

**Table 1.** *In vitro* Brine Shrimp lethality activity and cytotoxic activity *Calvatia gigantea extract* on Vero Cell Line.

	CC_50_ µg/ml	LC_50_ µg/ml
Test	Vero cell line (MTT assay)	Brine shrimp
*Calvatia gigantea*	>1000	1255.45
Cyclophosphamide	8.7 ± 0.2	–

**Figure 2.**
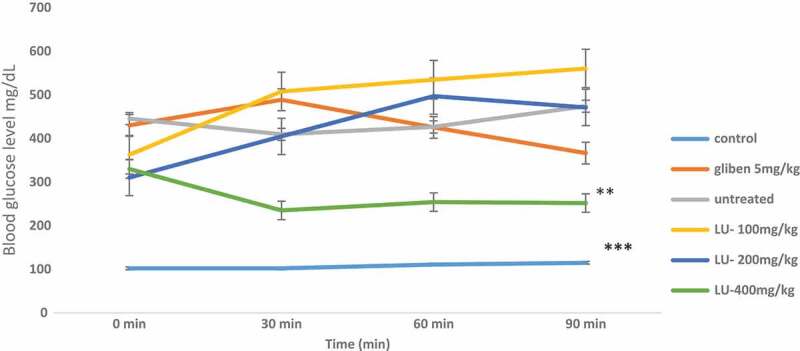
Effect of *Calvatia gigantea* on alloxan- induced diabetic rats within 90 min.

**Figure 3.**
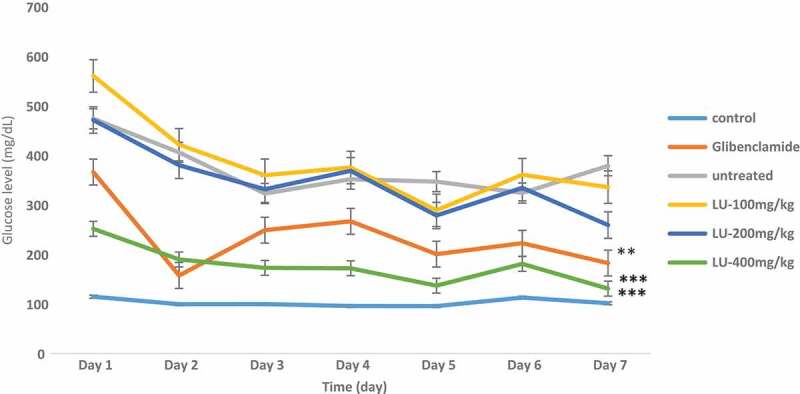
Effect of a *Calvatiagigantea*crude extract on fasting glucose level in alloxan – induced diabetic rats from day 1 to day 7.

The acute *in vivo* antidiabetic assay model, designed to assess the effect of the extracts on diabetic rats over a short period of time revealed that the 400 mg/kg of the extract at the 30th minute significantly reduced the blood glucose level from 330 mg/dL to 235 mg/dL that is by 28%. As reported by Priyanka *et al.*, 2010 a 25% reduction in blood glucose levels is considered a significant hypoglycemic effect, this assertion has also been confirmed by other authors (Sathya et al. 2008; Murthy et al. 2013; Xing et al. 2015). Furthermore, at the end of the 90th minute, there was a significant reduction of glucose level for all the doses of extract comparable to the standard glibenclamide (Figure 2). For the chronic model (7 days) *in vivo* assay, on day 3 there was a reduction in the blood glucose level for all the doses tested but this was more significant for glibenclamide (30%). At the end of the experiment, there was a significant reduction in the glucose level of all the groups, but this was more significant is the 200 mg/kg group (Figure 3).

**Key: LU 100mg/kg = ***Calvatia gigantea* extract 100mg/kg; **LU 200mg/kg = ***Calvatia gigantea* extract 200mg/kg; **LU 300mg/kg = ***Calvatia gigantea* extract 300mg/kg, *** = P < 0.0001, ** = P < 0.05

**Key: LU 100mg/kg = ***Calvatia gigantean* extract 100mg/kg; **LU 200mg/kg = ***Calvatia gigantean* extract 200mg/kg; **LU 300mg/kg = ***Calvatia gigantean* extract 300mg/kg. *** = P < 0.0007, ** = P < 005

**Figure 4.**
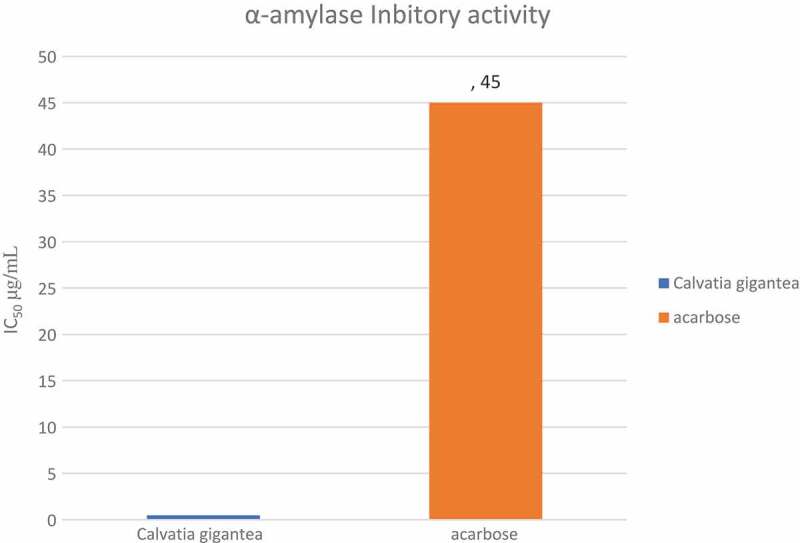
IC_50_ valuesof crude extract of a *Calvatia gigantea* and acarbose in α-amylase inhibitory assay.

**Figure 5.**
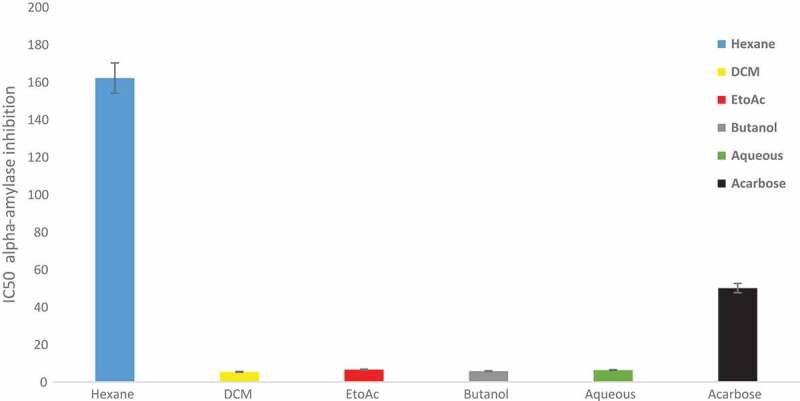
α-amylase inhibitory assay IC_50_ values of hexane, dichloromethane, ethylacetate, butanol and aqueous fractions of *Calvatia gigantea.*

The *in vitro* alpha amylase inhibitory assay indicated that the mushroom was 90 times more active than acarbose the standard drug. *C. gigantea* could inhibit 50% of the enzyme at 0.46 µg/mL while acarbose inhibited at 45 µg/mL (Figure 4). All the fractions (dichloromethane, ethyl acetate, butanol and aqueous) of *C. gigantea* possessed better α-amylase inhibitory activity than acarbose (IC_50_ 0f 5.3 µg/mL, 6.5 µg/mL, 5.7 µg/mL and 6.2 µg/mL respectively) except for the n-hexane fraction with an IC_50_ of 163 µg/mL (Figure 5). These findings suggest that *C. gigantea* might be a promising source of medicine and has the potential to be a health food and food supplementary product. In addition, the extract and its fractions may be explored for the development of natural product-based pharmaceutics.

## Conclusion and recommendation

The *in vitro* alpha amylase inhibitory assay established that the extract possessed potent antidiabetic activity. However, the observed reduction in antidiabetic activity upon fractionation of the extract demonstrates that the antidiabetic activity may reside in the whole plant and not in the fractions. The significant inhibition of the enzyme α-amylase and the *in vivo* antidiabetic activity exhibited by the mushroom investigated underscores the fact that the fungi could potentially serve as a functional food, as well as a drug lead.
